# RAD50 missense variants differentially affect the DNA damage response and mitotic progression

**DOI:** 10.1002/1873-3468.70175

**Published:** 2025-10-01

**Authors:** Hanna Redeker, Swantje Kebel, Lea Völkening, Anna Vatselia, Louisa Weinhold, Girmay Asgedom, Axel Schambach, Detlev Schindler, Thilo Dörk, Kristine Bousset

**Affiliations:** ^1^ Gynaecology Research Unit Hannover Medical School Germany; ^2^ Institute of Experimental Hematology Hannover Medical School Germany; ^3^ Institute of Human Genetics, Biocenter University Würzburg Germany

**Keywords:** breast cancer, DNA damage response, epirubicin, missense variants, MRN complex, RAD50 deficiency, separation of function

## Abstract

RAD50 is the central protein of the MRN complex and crucial in DNA double‐strand break repair. RAD50 deficiency causes a genomic instability disorder characterized by microcephaly and stunted growth. Using lentiviral constructs, we investigated whether cancer‐related RAD50 missense variants can complement the delayed damage response after exposure to the chemotherapeutic agent epirubicin and/or mitotic progression in RAD50‐deficient fibroblasts. Eight missense variants, all capable of forming an MRN complex, supported the DNA damage response and mitotic features to different extents, indicating these functions are separable. Three variants showed both an impaired epirubicin response and slowed cell division in the likely pathogenic range. Assessing RAD50 missense variants with distinct functional readouts may help to further elucidate their differential roles in immunodeficiency and cancer and could improve therapeutic strategies.

**Impact statement**

RAD50 has a strong impact on DNA repair and cancer therapy. Here, we analyse RAD50 missense variants at four functional levels. Some variants showed an impaired epirubicin response and mitotic progression in the pathological range, while for others these endpoints were separable. Functional heterogeneity of RAD50 variants could contribute to clinical variability.

## Graphical abstract

RAD50 incorporates into the MRN complex and initiates the DNA damage response. Furthermore, RAD50 promotes mitotic progression. RAD50 missense variants capable of forming an MRN complex supported the DNA damage response and mitotic features to different extents in complementation experiments, indicating these functions are separable and might impact clinical features of RAD50 deficiency and on the prognosis of cancer patients.

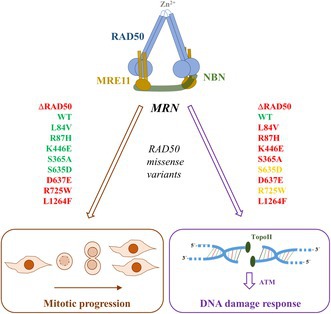

## Abbreviations

(N)CI (normalized) cell index

ANOVA analysis of variance

DSB double‐strand break

GVGD grantham variation and grantham deviation

MRN MRE11/RAD50/NBN complex

NBS Nijmegen breakage syndrome

NBSLD NBS‐like disorder

NHEJ nonhomologous end‐joining

TopoII topoisomerase II

Human RAD50 deficiency is a very rare autosomal recessive genomic instability disorder characterized by microcephaly and stunted growth [1, 2, 3, 4, 5]. These features are similar to the Nijmegen Breakage Syndrome (NBS) and have prompted the term NBS‐like disorder, NBSLD [1]. While most described patients did not show immunodeficiency [1, 2, 5], pathogenic missense or in‐frame deletion variants were described in two patients with anemia and immunological problems [3, 4]. Heterozygous *RAD50* mutational status may be associated with breast cancer risk in the Finnish population [6, 7] but large case–control studies have not confirmed an overall association of *RAD50* germline variants with breast cancer risk [8, 9, 10, 11]. However, RAD50 loss‐of‐function has repeatedly been linked with resistance to cytostatic agents [12, 13, 14, 15, 16, 17, 18, 19, 20], and RAD50 is regarded as one of the major contributors to cancer resistance toward chemotherapeutic drugs [21]. Therefore, *RAD50* as well as *MRE11* (encoding its binding partner) have been proposed as prognosis‐significant DNA repair genes to establish prediction models in breast cancer patients [22]. At present, RAD50 status is not routinely assessed, and a full implementation of *RAD50* genotyping for clinical research and prognosis would benefit from a more extensive characterization of missense variants with appropriate functional endpoints.

RAD50 interacts with MRE11 and NBN to form the MRE11‐RAD50‐NBN (MRN) complex. The MRN complex is recruited to DNA double‐strand breaks where it replaces the Ku complex at an early stage in the DNA damage response [23, 24, 25, 26]. RAD50 tethers broken DNA ends while MRE11 performs initial resection, and NBN recruits the ATM kinase to trigger multiple phosphorylation events that determine further steps of repair and, ultimately, cell fate [26]. This includes cell cycle arrest at the G2/M stage to prevent damaged cells from entering mitosis [27]. On the other hand, RAD50 also promotes timely mitotic progression in apparently unperturbed cells that have successfully entered M phase [28]. It is uncertain how the latter relates to its role in DNA repair, but since chemical inhibition of ATM had little effect and RAD50 phospho‐mutants are fully proficient in mitotic progression, the role in cell division appears to be largely independent of ATM kinase activity [28].

Consequences of amino acid substitutions are not easy to predict because they can affect several properties including protein stability, protein localization, post‐translational modification, protein interaction, or enzymatic activities. Structure‐guided prediction tools can identify missense variants that likely disturb MRE11 binding [29, 30, 31], dimerization, and ATPase‐related functions [25, 32, 33]. Investigations of RAD50 missense variants have shed light on the role of the RAD50 ATPase domain in the sensitivity to DNA damage and MRE11 nuclease activation and provided evidence for the importance of the Zn‐hook and coiled‐coil domain for RAD50 functionality [32, 33, 34, 35, 36, 37]. These studies have been performed in yeast and mice, while models to analyze RAD50 missense variants in human cells are largely lacking.

In this study, we establish cell culture methods to experimentally study the effect of specific RAD50 variants at four different levels: (i) RAD50 protein expression and MRN complex formation; (ii) intracellular localization; (iii) DNA damage response toward epirubicin, and (iv) mitotic progression. We propose that certain missense variants affect DNA damage response and/or mitosis despite normal RAD50 protein levels and MRN complex formation.

## Materials and Methods

### Cell culture and treatments

Large T‐immortalized fibroblast cells, derived from two patients with RAD50 deficiency, F239‐T [1, 28] and F583‐T [2], and from a healthy donor, ADD‐T [1, 2, 28], have been previously described [1, 2, 28]. F239‐T and F583‐T express very little RAD50 protein (less than 10% of wild‐type) and thus are representative of RAD50 loss‐of‐function, thereby providing a system to express RAD50 variants of interest by lentiviral transduction as previously described [28]. All cells were cultured in DMEM high glucose, 10–15% fetal bovine serum, 500 U·mL^−1^ penicillin, 0.5 mg·mL^−1^ streptomycin, and 2 mm L‐glutamine at 37 °C at 5% CO_2_. Epirubicin (Toronto Research Chemicals via Biozol, Hamburg, Germany) was dissolved to 1 mm in 150 mm NaCl, pH 5.5. Cisplatin (Sigma‐Aldrich, St. Louis, Missouri, USA) was resolved to 1 mm in 150 mm NaCl, pH 5.5, and olaparib (Biozol, LCL) was resolved in DMSO to 1 mm, respectively. Both agents were administered at a final concentration of 5 μm. Control cultures were treated with solvent only.

The experiments were undertaken with the understanding and written consent of each subject, and the study methodologies conformed to the standards set by The Code of Ethics of the World Medical Association (Declaration of Helsinki). The project was part of the work on genomic instability disorders approved by the Institutional Review Boards of the Faculty of Medicine at the University of Würzburg and at Hannover Medical School.

### Plasmids and cloning

Lentiviral plasmids and *RAD50* lentiviral vector generation have been described before [28]. Selected *RAD50* missense variants were cloned by overlapping PCR [38] using Q5 Hot Start High‐Fidelity DNA Polymerase (NEB) and verified by Sanger sequencing. Primer sequences are available upon request.

### Immunoprecipitations

For immunoprecipitations, cells were lysed in lysis buffer [1]. 25 μL slurry of Pierce protein A and G magnetic beads was washed with PBS‐0.02% Tween and incubated with 6 μL RAD50 antibody (ab89) in PBS‐0.02% Tween for 10 min at RT and 1 h at 4 °C in an overhead shaker. After washing, 600 μg protein was added in 600 μL lysis buffer and incubated for 10 min at RT and 1.5 h at 4 °C. After washing with PBS, beads were resuspended in SDS sample buffer and heated (5 min, 95 °C) before electrophoresis.

### SDS/PAGE and western blotting

SDS/PAGE and western blotting were performed as previously described [1, 2]. The following primary antibodies were used: anti‐RAD50, ab89, Abcam; anti‐NBN, GTX103229, Genetex; anti‐ACTB, A5541, Sigma.

### Immunofluorescence

Immunofluorescence was performed as described [39], except fixation with 3% formaldehyde/2% glucose in PBS, using anti‐RAD50 antibody (ab89; 1 : 150), anti‐mouse Alexa 488 antibody (A11018, 1 : 200; Invitrogen, Waltham, MA, USA), and DAPI (Invitrogen; 0.6 μm). Microscopy was performed on a ZEISS LSM980 with Airyscan 2 (MHH Research Core Unit Laser microscopy), using leica application suite advanced fluorescence (v1.9.0) software, zen 3 (blue edition) software, and fiji/imagej2 for visualization and analysis.

### xCELLigence

Cells were monitored after epirubicin treatment using impedance measurement with the xCELLigence RCTA technology (Agilent; https://www.agilent.com/en/technology/cellular‐impedance). In principle, adherent cells are seeded into specialized plates equipped with gold electrodes in the bottom of each well. While the cells are cultured, an electric field is applied and the current is monitored over time. Cells covering the electrodes impede the current, depending on their size, shape, attachment to the plate, and mobility. From the impedance measurement, a cell index (CI) is calculated and plotted as a function of time [40, 41].

In order to receive a baseline, 100 μL medium was added to Acea 96‐well Eview plates (OLS), equipped with embedded gold electrodes and placed into the xCELLigence device for baseline measurement. 2500–3000 cells per well in 100 μL medium was added and incubated for 30 min before measurement started. Epirubicin was added after ~24 h. Impedance measurements were taken every 15 min before adding epirubicin, and during the first 24 h of treatment either every 5 min or every minute, followed by 15‐min intervals. Cells were analyzed for 3 days and examined by microscopy before epirubicin administration and at the end of the experiment. Each condition was performed in quadruplicates. Data were analyzed and plotted by the rcta software. Cell indices (CI) were normalized to the last time point before treatment (*t*_epi‐0_). This normalized cell index (NCI) was then compared between cell lines and treatment conditions.

### Live cell imaging

Live cell imaging was performed using time‐lapse microscopy as previously described [28] in six‐well plates using a Leica DMI 6000B equipped with Incubator BL for providing 37 °C and 5% CO_2_. Imaging started 1 h after inhibitor or mock treatment for analyzing mitotic entry. Images were acquired every 5 min for 72 h (phase‐contrast optics, 20× objective (L40 × PH2)) and exported as time‐lapse movies.

### Bioinformatic prediction and statistical analysis

Prediction of pathogenicity for selected missense substitutions was performed using three prediction tools: AlphaMissense [42], ESM1b [43], and GVGD [44]. AlphaMissense provides a pathogenicity score between 0 and 1 as output (https://zenodo.org/records/8208688). ESM1b provides scores between 0 and −20, with more negative values indicating a higher likelihood of pathogenicity (https://huggingface.co/spaces/ntranoslab/esm_variants). The GVGD classifier provides seven grades (C65, C55, C45, C35, C25, C15, and C0), from the most likely deleterious C65 to the least likely deleterious C0 [44]. None of the patient‐derived missense variants enhanced alternative splice sites according to SpliceAI predictions (https://spliceailookup.broadinstitute.org/) [45].

Data from xCELLigence and mitosis experiments were analyzed using two‐way ANOVA with experiment as a covariable, and *P* < 0.05 was considered evidence of difference. Time‐lapse microscopy results of mitotic counts were analyzed using three‐way ANOVA in epirubicin‐treated and untreated strata, respectively, with cell line as the predictor variable and with experiment and 24‐h time window as covariable. Numbers of mitoses in treated and untreated ADD‐T and F239‐T cells, respectively, were also compared for each of the two experiments by Fisher's exact test, with a two‐tailed *P* < 0.05 considered evidence of difference.

## Results

### Selection and expression of cancer‐related RAD50 missense variants

**Fig. 1:**
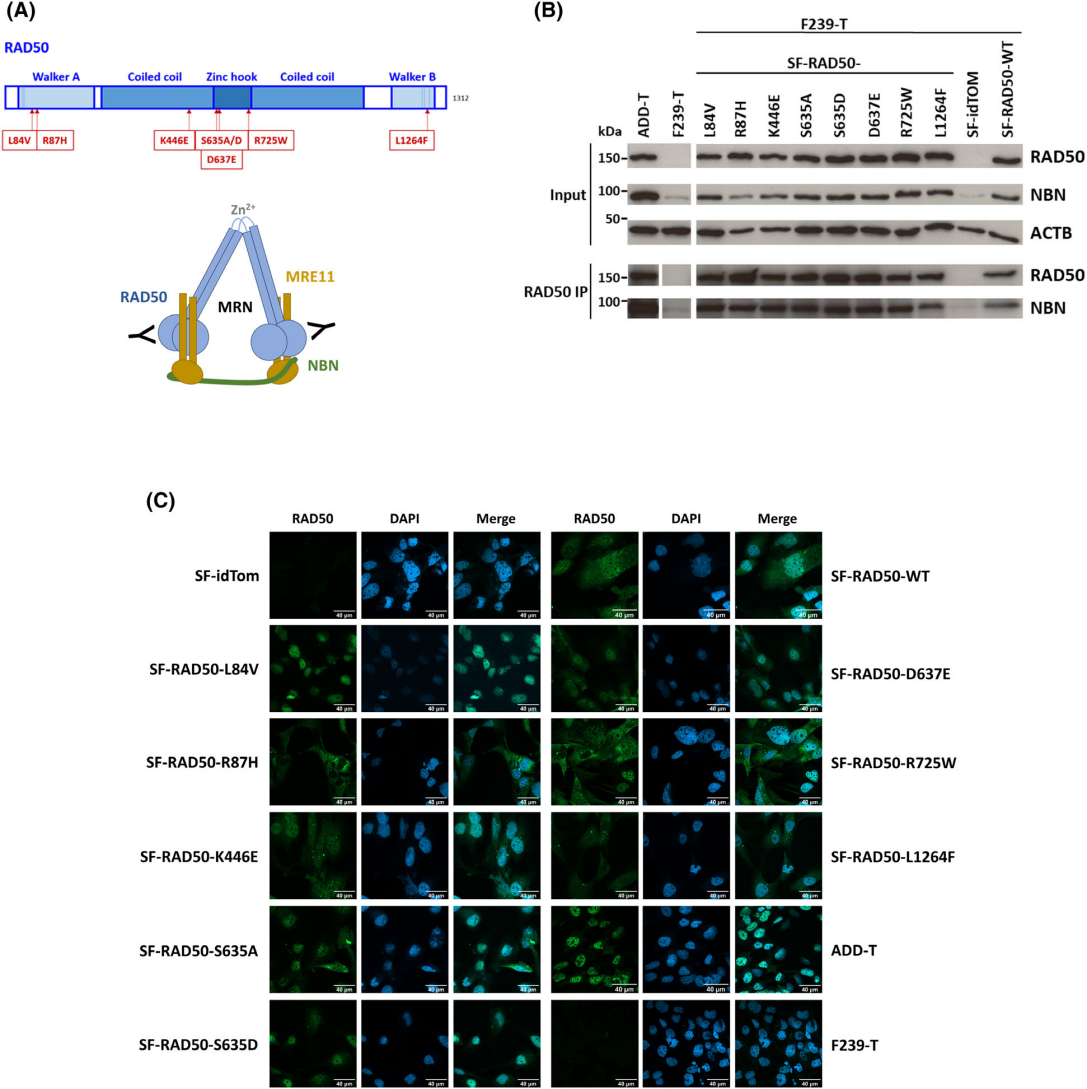
RAD50 variant distribution, incorporation into the MRN complex, and intracellular localization. (A) Schematic drawing of RAD50 indicating the location of the selected missense substitutions, and of the MRN complex (modified from [47]) with immunoprecipitation (IP) antibodies indicated. (B) Western blot analysis of RAD50 and NBN levels in cells expressing selected RAD50 missense variants. Input levels and the levels after RAD50 co‐immunoprecipitation are shown. The presence of NBN served as indication for MRN complex integrity. Actin B (ACTB) served as loading control for the input. (C) Intracellular localization of RAD50 variants by immunocytochemistry and confocal microscopy when expressed exogenously. RAD50‐proficient (ADD‐T) and the RAD50‐deficient parental cells (F239‐T) are shown for comparison. Scale bars of 40 μm length are displayed. To indicate vector control cells, the name of the lentiviral vector (SF‐idTom) was used for the transduced cell line (F239‐T). Similarily, the name of the lentiviral vector (SF‐RAD50‐variant) was used to indicate exogenous lentiviral expression.

For analyzing the impact of different RAD50 variants on specific RAD50 functions, we selected six RAD50 missense variants that had been associated with breast cancer in an early study [46] (Table 1, Fig. 1A). The predicted pathogenicity of these missense variants by Align GVGD, ESM1b, and AlphaMissense, and their ClinVar classification are listed in Table 1. Except for RAD50*p.L84V, all variants were listed in ClinVar and were recorded with “conflicting classifications of pathogenicity,” indicating a need for further characterization. AlphaMissense predicted RAD50*p.K446E and RAD50*p.L1264F as likely pathogenic, while RAD50*p.L84V and RAD50*p.R725W had the highest pathogenicity scores in ESM1b, and RAD50*p.K446E and RAD50*p.R725W had the highest pathogenicity scores in GVGD (Table 1). For comparison, and to further investigate the dependency on ATM signaling, we also included the phospho‐minus and phospho‐mimic variants RAD50*p.S635A and RAD50*p.S635D, respectively, in this study [28]. While RAD50*p.S635A was predicted as likely benign, RAD50*p.S635D was predicted to be likely pathogenic by AlphaMissense (Table 1), and ESM1b scores were inconspicuous for both substitutions. However, these prediction tools do not integrate knowledge of ATM phosphorylation sites.

**Table 1:** Features and predicted pathogenicity of selected RAD50 missense variants. Genomic coordinates refer to GRCh38.p14, NC_000005.10. Position of mRNA variant refers to isoform NM_005732.4. MAF, minor allele frequency according to the GnomAD database (https://gnomad.broadinstitute.org/gene/ENSG00000113522?dataset=gnomad_r4, accessed Jan 14, 2025).

Missense	Genomic	mRNA variant	Codon change	ESM1b	AlphaMissense	GVGD	ClinVar	MAF (GnomAD)	rsID
variant	coordinates		in construct	prediction	prediction	prediction	classification		
p.L84V	g.132575813C>G	c.250C>G	CTG>GTG	−9.42	0.230	C25	Not listed	6.24e‐7	rs2149836375
p.R87H	g.132575823G>A	c.260G>A	CGG>CAC	−8.54	0.092	C25	Conflicting	3.17e‐4	rs374561375
p.K446E	g.132589721A>G	c.1336A>G	AAG>GAG	−8.69	0.778	C55	Conflicting	3.11e‐4	rs149217423
p.D637E	g.132594986T>A	c.1911T>A	GAC>GAA	−6.41	0.393	C35	Conflicting	1.27e‐4	rs144749616
p.R725W	g.132595776C>T	c.2173C>T	CGC>TGG	−9.62	0.286	C65	Conflicting	5.02e‐5	rs369560280
p.L1264F	g.132642215C>T	c.3790C>T	CTG>TTC	−7.42	0.607	C15	Conflicting	6.81e‐5	rs199579239
p.S635A	g.132594978AG>GC*	c.1903AG>GC*	AGC>GCC	−5.92	0.170				
p.S635D	g.132594978AG>GA*	c.1903AG>GA*	AGC>GAC	−5.55	0.700				

* These two phosphorylation site mutations were neither drawn from clinical reports nor listed in ClinVar. A related missense substitution p.S635N is listed in dbSNP: rs2149843742, and in ClinVar: 1494466 (uncertain significance).

All eight *RAD50* missense variants were introduced into lentiviral constructs, followed by transduction of the RAD50‐deficient fibroblast line F239‐T. This patient‐derived cell line has strongly reduced levels of endogenous RAD50 protein [1, 28]. Therefore, this system allowed us to analyze to what extent the RAD50 variants of interest could complement specific RAD50 functions.

By immunoblotting, we confirmed that all RAD50 missense mutants tested were highly expressed (Fig. 1B). Furthermore, NBN was pulled down with all RAD50 mutants in co‐immunoprecipitation experiments (Fig. 1B), suggesting their proper folding and incorporation into the MRN complex. By immunocytochemistry, most of the RAD50 variants were predominantly found in the nucleus, but RAD50*p.R87H was predominantly found in the cytoplasm. A heterogeneous staining pattern was observed for RAD50*p.R725W, where cells with predominantly nuclear and cells with predominantly cytoplasmic protein were detected, and about equal cytoplasmic and nuclear staining was seen for RAD50*p.L1264F (Fig. 1C). These results indicated that some of the RAD50 missense variants may at least partially affect intracellular localization.

### Complementation analysis after epirubicin administration

**Fig. 2:**
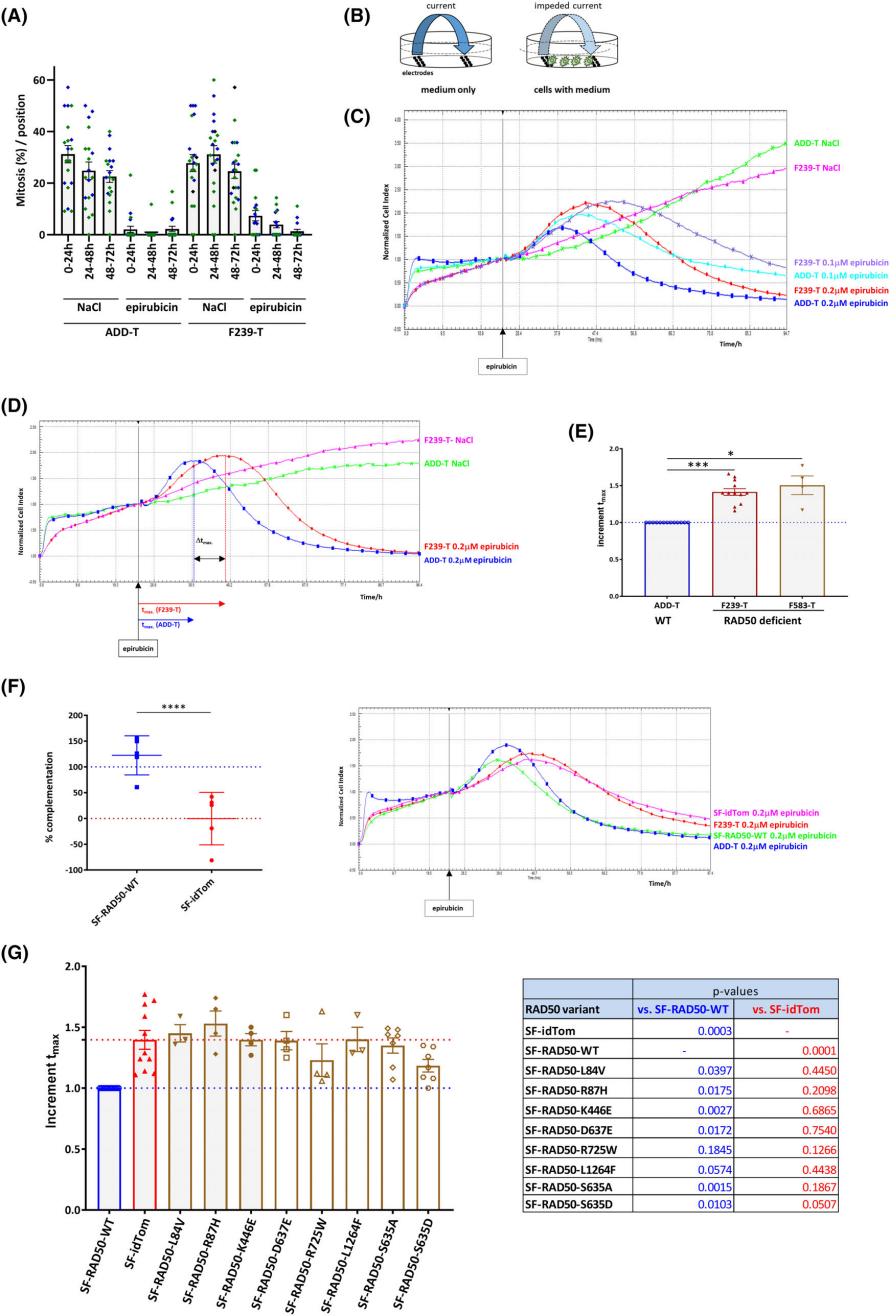
Monitoring epirubicin response with time‐lapse microscopy and xCELLigence. (A) RAD50‐proficient (ADD‐T) and RAD50‐deficient (F239‐T) cells were treated either with 0.2 μm epirubicin or with solvent (NaCl) only and monitored by live cell imaging for 3 days for entering a successful mitosis within the time intervals 0–24 h, 24–48 h, or 48–72 h post epirubicin administration, respectively. The fractions of cells entering a successful mitosis are presented relative to the initial cell number within each time interval. Data from two independent experiments with 8–13 microscopic fields evaluated are shown in blue and green, respectively, with bars indicating the mean and SEM. (B) Principle of xCELLigence measurement. Adherent cells were seeded in quadruplicates into 96 well plates with in‐ground gold electrodes. The current between the electrodes is monitored over time (e.g., every 2 or 5 min) and is impeded by covering cells, depending on their size, shape, attachment to the plate, and mobility. A cell index (CI) is calculated from the measured impedance and plotted as function of time. For the normalized cell index (NCI) the CI is related to the index at a defined time *t*_epi‐0_ (e.g., epirubicin administration) [41]. (C) Dose‐dependent response toward epirubicin in RAD50‐proficient (ADD‐T) and RAD50‐deficient (F239‐T) fibroblasts. At 24 h after seeding, cells were treated with either 0.1 μm or 0.2 μm epirubicin, respectively, and NaCl as solvent for 3 days and were monitored. A higher epirubicin dose resulted in earlier curve maximum and subsequent decline for both cell lines. (D) Introducing *t*_max_ to analyze the response toward epirubicin in RAD50‐proficient (ADD‐T) and RAD50‐deficient (F239‐T) fibroblasts from impedance measurement. The experiment was performed as in C, but with only 0.2 μm epirubicin. RAD50‐deficient cells (F239‐T) responded with delay to epirubicin when compared to WT cells. The time in epirubicin until maximum NCI was determined as *t*_max_; the delay for the mutant cell line compared to wild‐type ADD‐T was defined as Δ*t*_max_ = *t*_max_(F239‐T)−*t*_max_(ADD‐T). Mean NCI tracings from quadruplicates are shown. RAD50 deficient F239‐T cells exhibited a delay in t_max_ of about 7–9 h (see also C). (E) RAD50‐proficient (ADD‐T, *n* = 12) and RAD50‐deficient (F239‐T, *n* = 12 and F583‐T, *n* = 4) fibroblast lines were treated with 0.2 μm epirubicin and monitored by the xCELLigence system. Increment *t*_max_ = *t*_max_(cell line)−*t*_max_(ADD‐T)/*t*_max_(ADD‐T). Mean and SEM are depicted. Significance was analyzed by two‐way ANOVA with experiment as covariable and is indicated for comparison to ADD‐T (WT cells). *P < 0.05; ***P < 0.001. (F) Range of *t*_max_ after complementation of RAD50‐deficient F239‐T cells with lentivirally expressed wild‐type RAD50 (designated SF‐RAD50‐WT) in comparison to empty vector (designated SF‐idTom). Shown are means and SD of technical quadruplicates from five independent experiments with 0.2 μm epirubicin. Statistical analysis was performed using ANOVA and Šídák's multiple comparison test (****P < 0.0001). There were significant differences with *P* < 0.0001 between SF‐idTom and SF‐RAD50‐WT; *P* = 0.0008 for both ADD‐T vs F239‐T and ADD‐T vs SF‐idTom; and *P* < 0.0001 for F239‐T vs SF‐RAD50‐WT, while no significant difference was observed between ADD‐T and SF‐RAD50‐WT (*P* = 0.818) as well as F239‐T and SF‐idTom (*P* > 0.999). One complemetation experiment with four technical replicates for each condition is also shown. (G) Degree of complementation for eight RAD50 missense variants (SF‐RAD50‐variant) on response to epirubicin (0.2 μm), illustrated as incremental increase in *t*_max_. Increment *t*_max_ = *t*_max_(SF‐RAD50‐variant)−*t*_max_(SF‐RAD50‐WT)/*t*_max_(SF‐RAD50‐WT). Mean and SEM are shown; *n* = 12 for SF‐RAD50‐WT (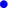); *n* = 11 for SF‐idTom (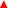); *n* = 7 for SF‐RAD50‐S635A (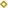) and SF‐RAD50‐S635D (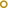); *n* = 4 for SF‐RAD50‐R87H (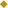) and SF‐RAD50‐R725W (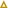); *n* = 3 for SF‐RAD50‐L84V (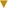), SF‐RAD50‐K446E (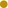), SF‐RAD50‐D637E (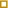) and SF‐RAD50‐L1264F (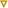). For each missense variant, the difference to both SF‐RAD50‐WT (blue) and SF‐idTom (red) were tested for significance using two‐way ANOVA with experiment as covariable. The respective *P* values are displayed in the accompanying table.

We then investigated whether the yet unclassified RAD50 variants were capable of supporting the intracellular DNA damage response after epirubicin exposure. The anthracycline epirubicin is a key component in the current treatment of breast cancer and acts as a topoisomerase II poison, promoting DSBs with covalently bound TopoII. MRN nuclease activity is critical for the removal of these DNA‐protein adducts and subsequent DNA repair [26, 48, 49, 50], making epirubicin exposure a valuable tool for monitoring the early RAD50 function in damage repair. Two initial time‐lapse microscopy experiments indicated two‐ to threefold higher residual mitotic activity within the first 72 h after epirubicin exposure of RAD50‐deficient F239‐T cells compared to wild‐type ADD‐T, suggesting that G2/M checkpoint activation is functional but less effective in the absence of RAD50 (Fig. 2A). To gain further insight into the time course of damage sensing, the cellular response to epirubicin was monitored over time using the xCELLigence system, which measures the impedance of a cell culture in real time and translates it into a cell index [40, 41] (Fig. 2B,C) depending on the number, size, and mobility of adherent cells. It has previously been shown that cytotoxic effects, especially by DNA‐damaging agents, lead to a characteristic impedance profile with a maximum of the cell index in a dose‐dependent manner and a subsequent cytotoxicity‐related decrease (Fig. 2C,D) [40, 41, 51, 52, 53].

We observed that RAD50‐deficient F239‐T cells required significantly longer time to reach the maximum cell index (*t*_max_) than WT cells (Fig. 2D). This delay in *t*_max_ was further confirmed in another RAD50‐deficient fibroblast line (F583‐T) [2] (Fig. 2E). This was an interesting outcome because the sensitivity of RAD50‐deficient cells toward other DNA‐damaging agents, cisplatin or olaparib, resulted in an earlier decline in the impedance profile (Fig. S1). Compared to cisplatin or olaparib, the mechanistically different compound epirubicin thus shifted the impedance time course toward the opposite direction. As the MRN complex is involved in removing TopoII adducts from DNA double‐strand breaks, followed by repair based on nonhomologous end‐joining (NHEJ) [26, 48, 49, 50, 54, 55], the delay in epirubicin response is consistent with impaired RAD50 function in this early damage recognition and processing step. Since epirubicin is the standard drug in breast cancer treatment and evoked this characteristic difference in RAD50‐deficient cells, we chose epirubicin response as a readout to further monitor RAD50 function.

Expression of RAD50‐WT lentiviral construct, but not the empty vector, rescued the timely response to epirubicin (Fig. 2F), indicating that this delay can be used to examine the ability of different RAD50 variants to cope with epirubicin‐induced DNA damage. Subsequent analysis of all RAD50 variants, including the phospho‐mutants, revealed that none fully complemented for RAD50, although two variants, RAD50*p.R725W and the phospho‐mimic RAD50*p.S635D, partially complemented (Fig. 2G). These results indicated that cancer‐related RAD50 missense variants, as well as the ATM phosphorylation site, impact the DNA damage response toward epirubicin.

### Effect of RAD50 missense variants on mitotic progression

**Fig. 3:**
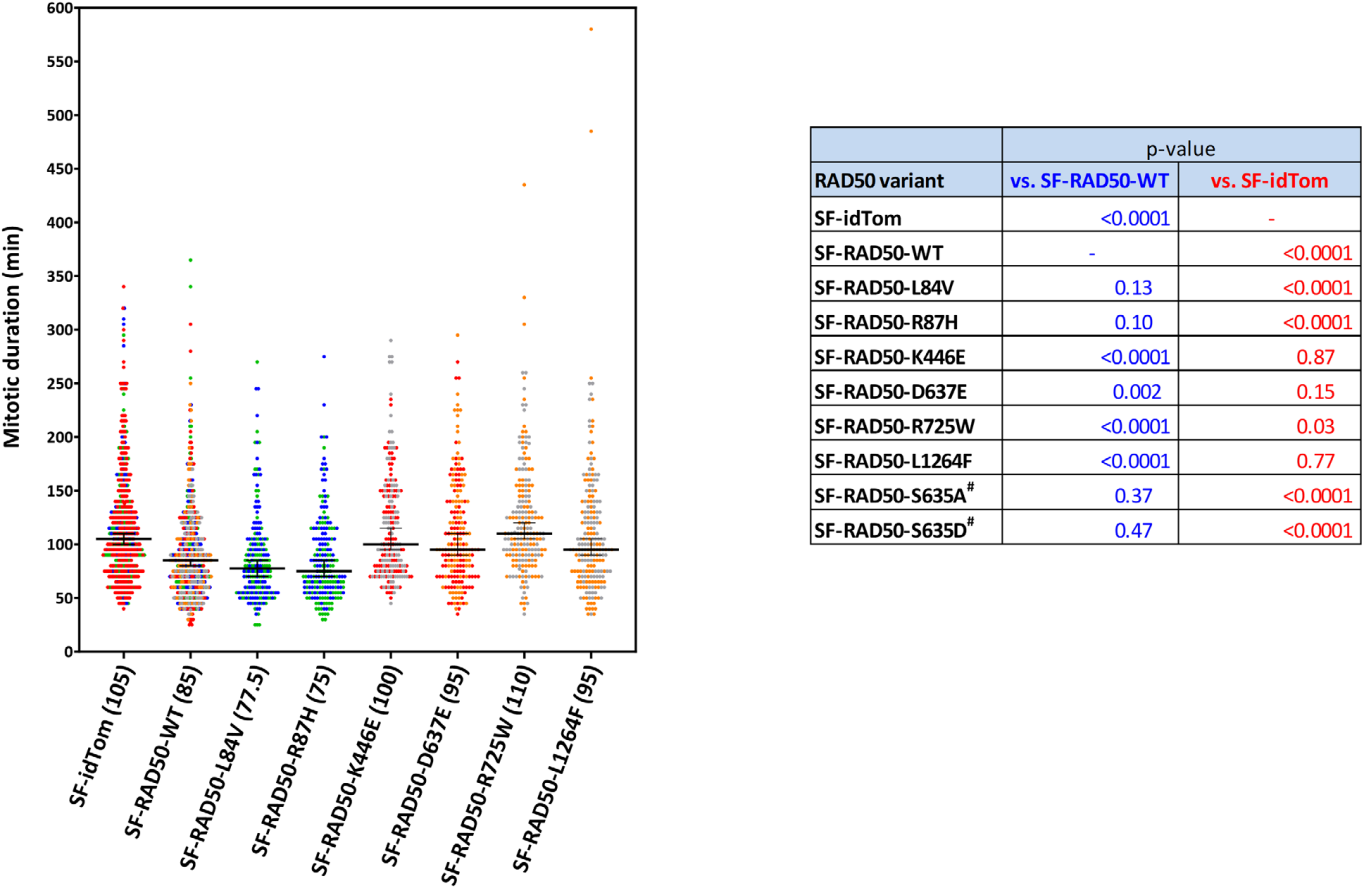
Impact of RAD50 missense variants on mitotic progression. Live‐cell imaging of F239‐T cells expressing either wild‐type RAD50 (termed SF‐RAD50‐WT), the respective SF‐RAD50 variants (SF‐RAD50‐variant) or the empty vector control (SF‐idTom) were monitored for 3 days. Mitotic duration was defined as the time from rounding up of a cell to settling down of the two daughter cells. 92–100 cells per variant and experiment were analyzed. The graph depicts data points for each cell line with their median (also in brackets at the *x*‐axis) and 95% confidence interval. The color of each value reflects the individual experiment. *P*‐values were determined by two‐way ANOVA with experiment as covariable are displayed, too.

We have previously observed that RAD50 promotes progression through mitosis, and this role seems to be independent of the ATM phosphorylation site, as p.Ser635 mutants exhibited normal mitotic progression [28]. We now sought to analyze the effect of the other selected RAD50 missense variants on mitotic duration by live cell imaging. Of the six patient‐derived RAD50 missense variants, RAD50*p.L84V and RAD50*p.R87H promoted mitotic progression similarly to RAD50‐WT. In contrast, all other variants showed a slow mitosis phenotype indicating that they could not complement RAD50 deficiency regarding this endpoint (Fig. 3). Taken together, these results provided functional support for the pathogenicity of the mutants investigated. However, some RAD50 variants (RAD50*p.L84V, RAD50*p.R87H, and RAD50*p.S635A) that were dysfunctional in response to epirubicin progressed normally through mitosis (Table 2).

**Table 2:** Summary and comparison of the effects exerted by the analyzed RAD50 missense variants. Some variants complemented individual aspects of RAD50 functionality to a different degree (full complementation indicated in blue, intermediate in black; poor complementation in red). RAD50 variants are termed after the vector used for their expression (“SF‐RAD50‐variant”), and SF‐idTom designates the vector control. WT stands for wild‐type, N and C stand for nuclear and cytoplasmic, respectively.

RAD50 variant	Localization	MRN complex	Epirubicin response	Mitosis
SF‐idTom	−	−	Delayed	Slow
SF‐RAD50‐WT	N>C	+	Normal	Normal
SF‐RAD50‐L84V	N>C	+	Delayed	Normal
SF‐RAD50‐R87H	N<C	+	Delayed	Normal
SF‐RAD50‐K446E	N>C	+	Delayed	Slow
SF‐RAD50‐D637E	N>C	+	Delayed	Slow
SF‐RAD50‐R725W	N/C	+	Intermediate	Slow
SF‐RAD50‐L1264F	N~C	+	Delayed	Slow
SF‐RAD50‐S635A	N>C	+	Delayed	Normal ^a^
SF‐RAD50‐S635D	N>C	+	Intermediate	Normal ^a^

^a^ Mitotic timing of the phospho‐minus and phospho‐mimic mutants was reported previously [28].

## Discussion

The classification of nontruncating RAD50 variants with unknown pathogenicity is clinically important but challenging and may incorporate functional exploration, *in silico* prediction, genetic association analyses and population frequency data. Adding to the complexity, separation‐of‐function variants could lead to specific cellular deficiencies and might modulate RAD50‐related clinical phenotypes. Such RAD50‐related phenotypes include different clinical characteristics in patients with the autosomal recessive syndrome RAD50 deficiency, such as immunodeficiency or cancer. They also include potentially mutation‐specific impacts on cancer risk and cancer prognosis. RAD50 has been described as a predictive factor for the success of chemotherapy in several different cancers, largely deduced from the analysis of *RAD50* transcript levels or the presence of loss‐of‐function variants [12, 13, 14, 15, 16, 17, 18, 19, 20, 21, 22]. The potential value of RAD50 missense variants is exemplified by a previous case report of synthetic lethality to DNA damage checkpoint inhibition in combination with DNA‐damaging chemotherapy in cancer cells harboring the RAD50*p.L1237F variant [13]. The role of *RAD50* variants for the risk of cancer, however, is less clear, and more case–control real‐life data are essential for the risk evaluation. In particular, breast cancer risk does not seem to be generally increased by truncating or missense RAD50 variants [8, 9, 10, 11], although certain substitutions such as the Finnish founder variant RAD50*c.687delT may confer some increased breast cancer risk [6, 7]. The latter frameshift variant would lead to a very early translational stop (p.Ser229Agfs*6) and is difficult to address experimentally because it is presently unclear whether any truncated RAD50 protein arises from this allele. Mere loss‐of‐function variants with very low RAD50 were represented in our study because we used two RAD50‐deficient lines, an empty vector as negative control, and a wild‐type construct as positive control. This sets the margins for testing the functional impact of defined missense substitutions with uncertain significance using lentiviral complementation. These variants, located in both globular ATPase domains, the coiled coils, and close to the zinc hook, were drawn from the same early breast cancer study [46] and, although several more variants have meanwhile been reported and tested for their association with breast cancer, they were taken as a pilot group of variants for functional testing.

We observed that some RAD50 functions were differentially affected by specific rare RAD50 missense substitutions. All analyzed variants were able to interact with NBN, suggesting they are capable of forming MRN complexes. Nevertheless, RAD50*p.R87H was mainly found in the cytoplasm, and RAD50*p.R725W and RAD50*p.L1264F showed a heterogeneous distribution, suggesting that their MRN complex formation might have been insufficient for nuclear transport. Aberrant intracellular localization is a known disease‐causing mechanism for a subset of pathogenic missense variants in several human disorders [56] and will need to be considered in RAD50‐related conditions as well. Interestingly, RAD50*p.R87H impaired epirubicin response but still complemented for mitotic delay despite its predominantly cytoplasmic localization, suggesting that RAD50*p.R87H may support mitotic progression after nuclear envelope breakdown.

MRN, especially MRE11 nuclease activity, is required for nucleolytic removal of TopoII‐DNA‐complexes [26, 48, 49, 50]. Slower recognition and/or resection by the MRN when harboring RAD50 missense variants likely causes the observed delay in epirubicin response. In addition to protein DNA adducts, compaction and incomplete tether breakage by trapped topoisomerase II might further impede DNA damage recognition [57] It is interesting to note that the epirubicin‐induced delay overrides the time‐to‐peak effect of opposite direction that usually accompanies the higher sensitivity of our RAD50 deficient cells toward double‐strand breaks (such as those induced by platinum or olaparib) related to impairment of ATM signaling and subsequent homology‐directed DSB repair. Thus, the functional deficiency in early TopoII‐DNA–complex removal had a particularly high impact on the time course of cell proliferation and survival. We have thus chosen epirubicin as a direct inducer of MRN functional activity, with the delay observed in RAD50 deficiency likely being proportional to the time needed for the processing of TopoII poisoned sites. Although their epirubicin response is significantly delayed, the mutant cells still followed the characteristic impedance profile induced by DNA‐damaging agents. Additional proteolytic and non‐proteolytic pathways to remove TopoII adducts may work as backup systems in MRN‐deficient cells, including Tyrosyl DNA‐phosphodiesterase 2 (TDP2), SPRTN, and VCPI/p97 [26, 50]. MRN was reported to be efficient in removing TopoII‐bound DNA, with very small levels still being sufficient and MRE11 deficiency showing a more severe phenotype than TDP2 loss in the presence of etoposide [49]. In this respect, it is important to consider that the RAD50 deficient cell line used for complementation in our experiments still expresses 5–10% background level of RAD50 [1] and may retain some normal epirubicin response.

Importantly, the roles of RAD50 in DNA damage response toward epirubicin and in promoting mitotic progression were separable, as we identified RAD50 variants supporting only the latter efficiently: RAD50*p.L84V, RAD50*p.R87H, and RAD50*p.S635A. While the differential effect of RAD50*p.R87H could be related to its cytoplasmic location, the effects of RAD50*p.L84V and RAD50*p.S635A appeared intrinsic to nuclear RAD50. The mutant RAD50*p.S635D was also proficient in mitotic duration and was intermediate in the epirubicin response. ATM phosphorylates RAD50 at this site upon genotoxic stress, including TopoII inhibition [58, 59], which may be mimicked by the Ser635Asp substitution resulting in some activity toward epirubicin treatment. In contrast, mitotic duration appears to be largely independent of the ATM kinase [28].

Our study also identified missense variants that were deficient in both epirubicin response and mitotic progression to a similar extent as RAD50‐deficient lines, thereby providing functional support of pathogenicity. These variants were RAD50*p.K446E, RAD50*p.D637E, and RAD50*p.L1264V, which were also the variants with the strongest *in silico* predictions, and – to a lesser extent – RAD50*p.R725W (Table 2). Such data may be important for aspects of RAD50 deficiency, a very rare genetic disorder, but they could also inform larger case–control studies of cancer in populations where the specific missense variants occur more frequently, for example, RAD50*p.L1264V in East Asians [60, 61].

Taken together, we describe RAD50 missense variants, previously identified in breast cancer patients, that support mitotic progression and DNA damage response toward TopoII poisoning to varying extents. Our data thus provide evidence for the functional diversity of RAD50 missense variants which may translate into a differential response to cancer treatment with therapeutics that cause DNA damage and/or cell division defects, respectively. Considering cancer risk, the differential impact of certain variants on DNA repair *versus* mitotic progression could be relevant for cancer development. Functional characterization of specific RAD50 missense variants would thus be important for the diagnosis of RAD50 deficiency, but also for better prediction of potential immunodeficiency, cancer risk, and cancer treatment.

## Author contributions

AS, KB, and TD conceptualization. LV, DS, and KB methodology. HR, SK, TD, and KB data curation and formal analysis. HR, SK, LV, AV, LW, GA, and KB investigation. KB writing original draft. KB, HR, DS, and TD writing – review and editing. TD funding acquisition. KB and TD supervison.

## Conflict of interest

The authors declare no conflict of interest. The supporting Claudia von Schilling Foundation did not influence the design, performance, and outcome of the study.

## Supporting information


**Fig. S1.** xCELLigence profile of WT and RAD50 deficient cells after administration of cisplatin and olaparib, respectively.

## Data Availability

Plasmid and primer sequences are available upon request (bousset.kristine@mh-hannover.de). Please see Fig. S1 for additional data.
