# Supplementary material for: RAD50 missense variants differentially affect the DNA damage response and mitotic progression
Source: FEBS Lett. 2025 Oct 1;599(24):3656–68. doi: 10.1002/1873-3468.70175 (PMC12720227; doi:10.1002/1873-3468.70175)
Supplement: Supplementary file 1 — Fig. S1. xCELLigence profile of WT and RAD50 deficient cells after administration of cisplatin and olaparib, respectively. [file FEB2-599-3656-s001.pdf]

Supplementary Figure S1

(A)

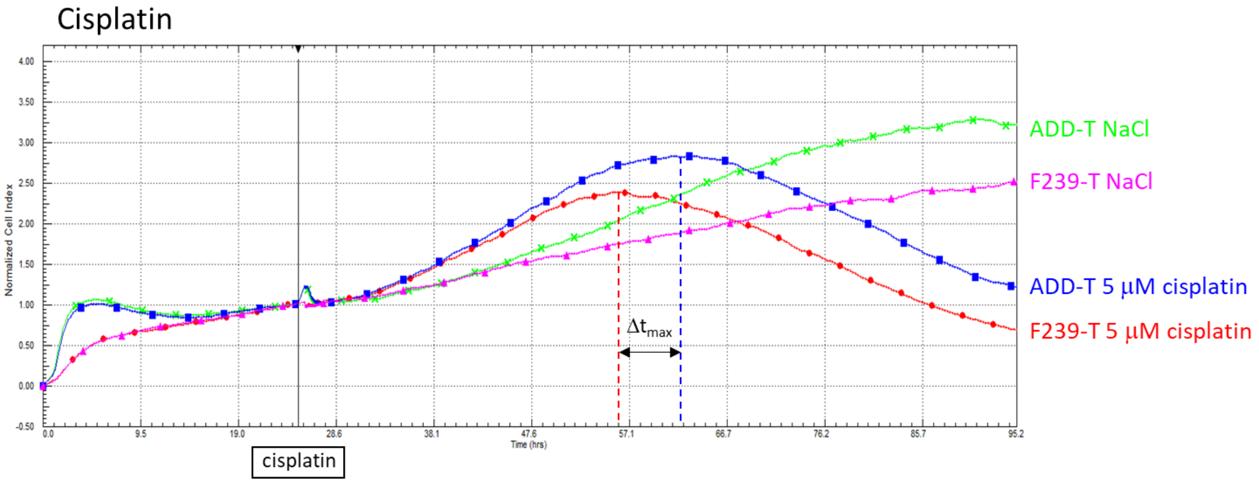

(B)

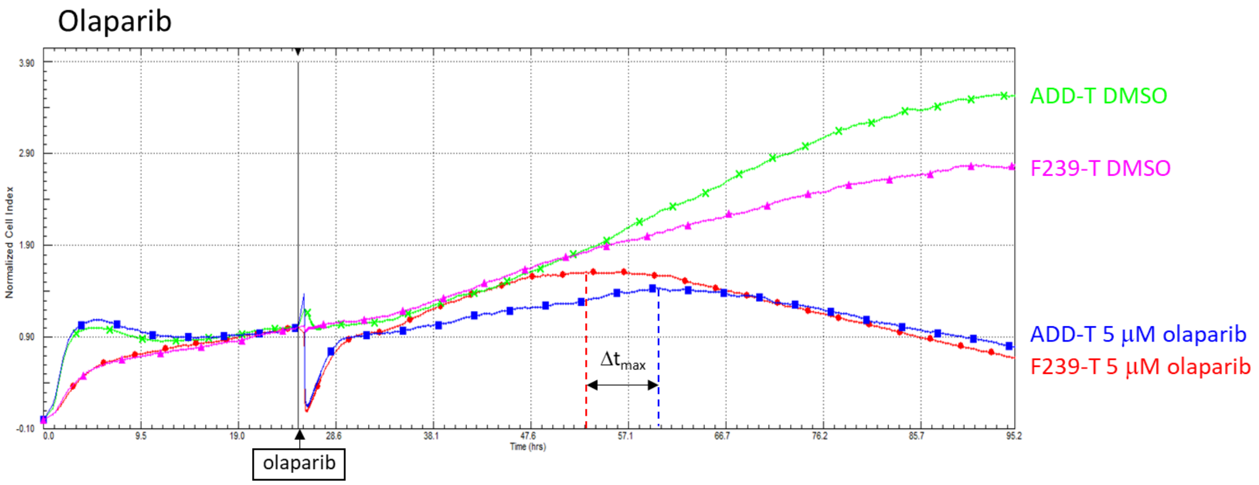

Supplementary Figure S1: xCELLigence profile of WT and RAD50 deficient cells after administration of cisplatin and olaparib, respectively.

ADD-T wildtype and F239-T RAD50 deficient fibroblasts were grown in specialized 96 well plates and monitored by the xCELLigence system for 24 hours before treatment. (A) Cisplatin was added at 5µM while control cultures were treated with solvent (150 mM NaCl) only. (B) The PARP inhibitor olaparib was added at 5µM while solvent only (DMSO) was added to control cultures. (A,B) Further monitoring was carried out for about 3 days. Each graph represents one experiment with the mean of four technical replicates. The time in epirubicin until the normalized cell index (NCI) reached a maximum, t_{max} (dashed lines), and the difference in t_{max} between RAD50 deficient and wildtype cells, Δt_{max} (arrow), are indicated for illustration.
